# Comparative analysis of non-linear growth model fit in chicken growth: a systematic review

**DOI:** 10.1007/s11250-026-05074-x

**Published:** 2026-06-17

**Authors:** Kholofelo Mogalanyane Thankge, Joas Albino Tsenane, Kwena Mokoena, Prudence Shibe Mokgapa, Madumetja Cyril Mathapo, Thobela Louis Tyasi

**Affiliations:** Department of Agricultural Economics and Animal Production, University of Limpopo, Private Bag X1106, Sovenga, Limpopo 0727 South Africa

**Keywords:** Body weight, *Gallus gallus*, Growth patterns, Weibull, Gompertz, Logistic

## Abstract

Estimation growth curve is a critical aspect of chicken production, influencing management decisions and optimization of growth rates. The poultry industry struggles to make effective decisions on feed management, growth rate, and slaughter age, which are key factors influencing productivity and profitability. Thus, the aim of this systematic review was to identify the most suitable non-linear model for estimating growth curves in chickens. A literature search was conducted using population, exposure, and outcome (PEO) framework. A total number of 49 articles were included in the review following PRISMA guideline and were published between 1995 and 2024. Databases such as Google Scholar, ScienceDirect, PubMed, and Web of Science, with the combination of the following keywords: chickens OR ‘*Gallus gallus*’ OR hens OR cocks OR chicks, AND ‘growth curve’ OR ‘growth patterns’ OR ‘growth parameters’, AND ‘non-linear models’ OR ‘non-linear mathematical models’ OR ‘non-linear statistical models’ were used. The results showed that Logistic model was the most used (n = 44) non-linear model, followed by Gompertz model (n = 43) and the von Bertalanffy model (n = 31). Gompertz, and Weibull models provided the best fit for estimation of chicken growth curves, with high coefficients of determination (R² = 1.00). The Gompertz, and Weibull non-linear models are recommended for estimating chicken growth curves to support breed selection, optimize slaughter age and reduce cost.

## Introduction

Chicken production plays a crucial role in developing countries by improving food security and to provide income, and it is a source of protein by providing meat and eggs (Mouffok et al. 2019). However, poultry industries face the critical challenge of making good decisions regarding feed supply and the cost for estimated daily gains and optimised slaughtering age in the production cycle, as these factors affect productivity and profitability (Quintana-Ospina et al. 2023; Gautam 2024). Growth curves can be used to describe change in live weight and body composition over time by employing non-linear models (Xie et al. 2020; Fatai et al. 2023). Non-linear models such Gompertz, Logistic, Richards and Brody have sigmoidal structure which has ability to capture biological growth information of animals (Topal and Bolukbasi 2008). Individual articles (Pinzon et al. 2022; Setiaji et al. 2023; Osaiyuwu et al. 2024) that are included were reporting on different models with different results. However, there was no systematic review that summarise information on the estimate growth curves of chickens. Thus, this systematic review is important to merge the articles’ results and give comprehensive conclusion on the best models to estimate growth curves of chickens. Therefore, it is essential to explore growth curves to dynamically understand and predict the growth and development of the chickens to maximise their production potential. The aim of this study was to provide information to discover the best non-linear model for prediction of growth curves. Implementing growth curve modelling in chicken production enhances farmer’s management skills to monitor animal health, optimizing slaughter age, evaluating selection program effectiveness, estimating daily feed intake.

## Materials and methods

### Eligibility criteria

The identification of Population, Exposure, and Outcomes (PEO) framework of the research question was done in this systematic review. The ‘chickens’ were referred as population, ‘non-linear models’ as the exposure, and the ‘best non-linear models to estimate growth curves of chickens’ as outcomes. Prior to conducting the study, a preliminary search of the PEO components was performed on databases, including Google Scholar, ScienceDirect, PubMed, and Web of Science.

### Search strategy

Databases such as Google Scholar, ScienceDirect, PubMed, and Web of Science, and the combination of the following keywords: chickens OR ‘Gallus gallus’ OR hens OR cocks, AND ‘growth curve’ OR ‘growth patterns’, AND ‘non-linear models’ OR ‘non-linear mathematical models’ OR ‘non-linear statistical models’ were used to conduct the search.

### Inclusion criteria

Articles were included if they focused on estimation of growth curves in chickens using non-linear model. Only articles published in English, and before year 2025 were considered.

### Exclusion criteria

Duplicates articles, articles that lack relevant keywords, research conducted on other species not chickens, articles not written in English, and review articles were removed.

### Data extraction

The extracted data was on chicken species, publication year, author, full in text written in English, and type of non-linear model.

## Results

### Search strategy

**Fig. 1:**
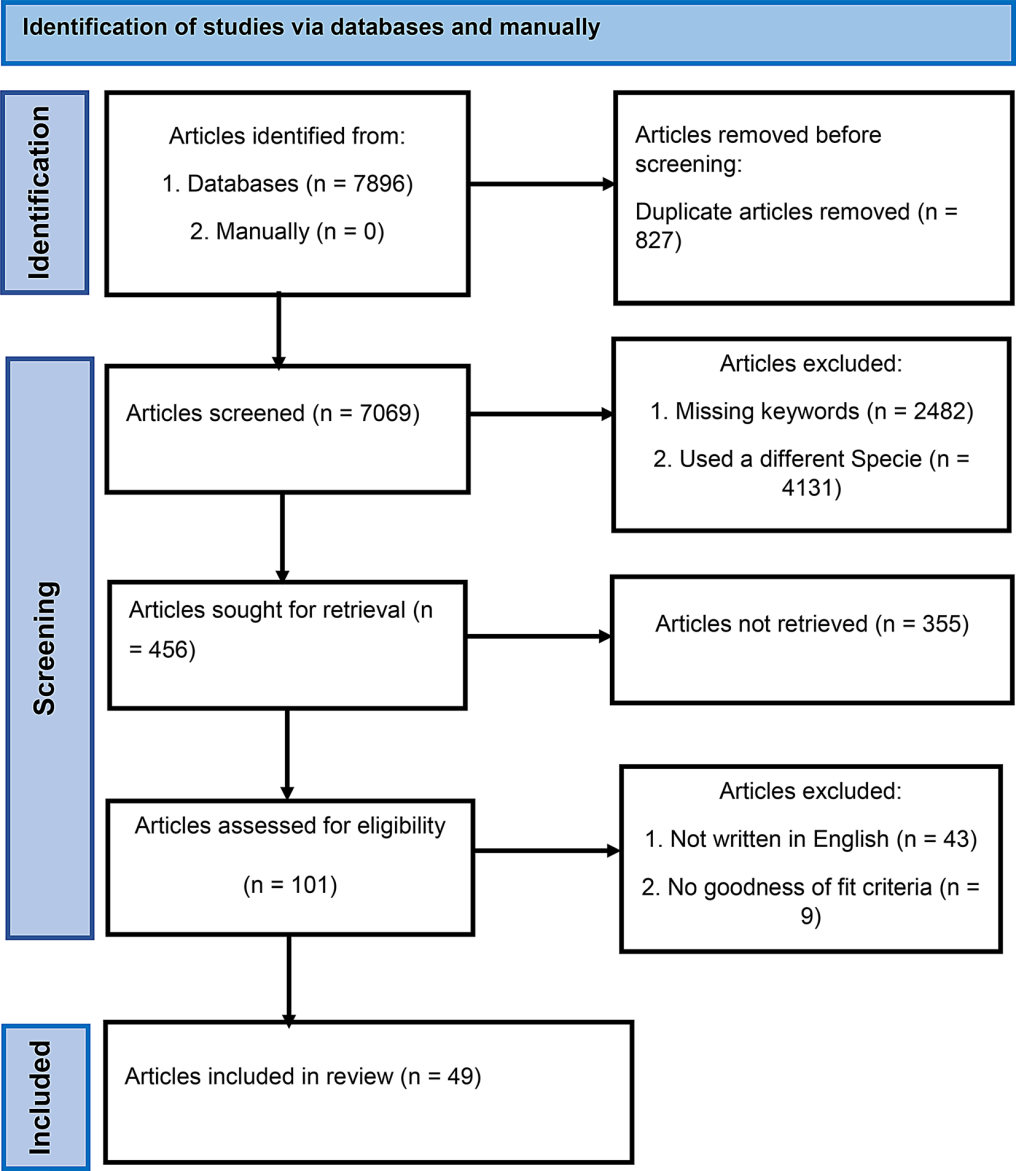
Flow chart of the process of study selection

Figure 1 shows PRISMA flow chart of identification and selection of articles used in the systematic review. The search retrieved 7896 articles, and from which 827 duplicates were excluded. After screening for eligibility, 7020 articles were excluded, and the remaining 49 articles met the criteria and were included in the review.

### Characterization of included articles

Table 1 shows the characterisation of articles included. The results indicated that out of 49 articles included in this review, Ghaderi-Zefrehei et al. (2024) used the largest sample size (*n* = 8334) of chickens. Galeano-Vasco et al. (2014) and Mata-Estrada et al. (2020) employed same sample size (*n* = 100) of chickens, furthermore, Pinzon et al. (2022) and van der Klein et al. (2020) used the same but smallest sample size (*n* = 15) of chickens. Indigenous chickens were the major chicken breeds studied by twenty-eight articles (*n* = 28). Nineteen (*n* = 19) countries were represented in 49 included articles published between 1995 and 2024.

**Table 1:** Characterisation of articles included

Authors	Year	Country	Journal	Breed	Age (days)	Sample size	Types of non-linear models
Aggrey	2002	United States	Poultry Science	Athens Canadian Random Bred	0-170	365	Gompertz, Logistic, Richards
Akinsola et al.	2021	Nigeria	Journal of applied animal research	Kuroiler, Sasso, Fulani, FUNAAB Alpha, Shika-Brown, Noiler	0-140	1939	Gompertz, Logistic,
Al-Samarai	2015	Iraq	American Journal of Applied Scientific Research	Ross 308	1–42		Gompertz, Logistic
Bashiru et al.	2020	Nigeria	Slovak Journal of Animal Science	FUNAAB Alpha	0–140	300	Gompertz, Logistic, von Bertalanffy, Richards
Benvenga et al.	2022	Brazil	AgriEngineering	Broilers			Gompertz, Logistic, von Bertalanffy
Bo et al.	2022	Vietnam	Journal of Animal and Plant Sciences	Ri chickens	0-140	318	Von Bertalanffy, Gompertz, Logistic, Richards
Boonkum et al.	2021	Thailand	Veterinary Science	Kai Shee, Kaimook e-san1	0–28, 28–140	180	Von Bertalanffy, Gompertz, Logistic
Brito et al.	2021	Brazil	Spanish Journal of Agricultural Research	Color Plume	0–77	432	Gompertz
de Figueiredo et al.	2023	Brazil	Livestock Science	Athens Canadian Random Bred	0-170		Brody, von Bertalanffy, Logistic, Gompertz, Richards
Falana et al.	2024	United States	SSRG International Journal of Recent Engineering Science	Cobb500	0–63		Gompertz, von Bertalanffy, Logistic
Faraji-Arough et al.	2019	Iran	Poultry Science	Khazak	0- 209	120	Gompertz, Logistic, von Bertalanffy, Richards
Fatai et al.	2023	Nigeria	Nigerian Journal of Animal Production	FUNAAB Alpha, Yoruba	0-168	256	Gompertz, Logistics, von Bertalanffy
de Freitas et al.	2023	Brazil	Acta Scientiarum Animal Sciences	Cobb500	1–42	1440	Gompertz
Galeano-Vasco et al.	2014	Colombia	Revista Brasileira de Zootecnia	Lohmann LSL layers	20–553	100	von Bertalanffy, Richards, Gompertz
Gautam	2024	India	Journal of Animal and Plant Sciences	Kadaknath	0–98	96	Logistic, Gompertz, von Bertalanffy, Richard
Ghaderi-Zefrehei et al.	2024	Iran	Veterinary Medicine and Science	Isfahan indigenous chicken	0–7, 8–56, 57–84	8334	Logistic, Gompertz, von Bertalanffy, Brody, Weibull
Quintana-Ospina et al.	2023	Colombia	Animals	Ross 308	0–35	2702	Logistic, von Bertalanffy
Knizetova et al.	1995	Czech	Genetics Selection Evolution	White Cornish, White Plymouth Rock	0-182	62	Richards
Kucuktopcu and Cemek	2021	Turkey	International Journal of Agriculture and Wildlife Science	Ross 308	0–40	20,035	Logistic, Richards, von Bertalanffy
Kumar et al.	2022a	India	Acta Scientific Veterinary Sciences	Aseel native chicken	0-140	1054	Gompertz, Logistic, von Bertalanffy
Kumar et al.	2022	India	The Indian Journal of Veterinary Sciences and Biotechnology	Aseel native chicken	84–140	247	Gompertz, Logistic, von Bertalanffy
Machado et al.	2023	Brazil	Brazilian Journal of Poultry Science	Canela-Preta	0-176	400	Logistic, Richards, von Bertalanffy, Gompertz,
Manjula et al.	2018	Korea	Asian-Australasian Journal of Animal Sciences	Korean native chicken	0-140	585	Gompertz, Logistic, von Bertalanffy
Masoudi et al.	2017	Iran	International Journal of Avian and Wildlife Biology	Ross 308	1–42	380	Logistic, Gompertz, Lopez, Richards and von Bertalanffy
Mata-Estrada et al.	2020	Mexico	Poultry Science	Creole	0-177	100	Logistic, Richards, von Bertalanffy
Michalczuk et al.	2016	Poland	Annals of Animal Science	Medium growing chickens	0–63	1000	Gompertz, Logistic, Richards
Moharrery and Mirzaei	2014	Iran	Journal of Animal and Feed Sciences	Ross 308, Iranian native chickens	14–56	144	Gompertz, Logistic, Lopez, Richards, Weibull
Mouffok et al.	2019	Algeria	Poultry Science	Cobb500	0– 49	50	Gompertz, logistic, Weibull, Richards, von Bertalanffy
Al-Ali et al.	2022	Iraq	Indian Journal of Ecology	Iraqi brown indigenous chickens	1-112	628	Gompertz, logistic, von Bertalanffy, Brody
Narushin and Takma	2003	Turkey	Biosystems Engineering	Shaver White laying hens	0–63		Gompertz, Logistic, Richards, von Bertalanffy, Weibull
Nematzadeh et al.	2021	Iran	Ankara Universitesi Veteriner Fakultesi Dergisi	Arian	0–42	823	Logistic, Gompertz, Richards, Von baretanalffy
Neysi et al.	2023	Iran	Animal Science	Fars indigenous chicken	0–84	15,222	Logistic, Gompertz, Brody, Weibull
Hoang et al.	2021	Vietnam	Animal Science	Mia chickens	0-140	224	Logistic, Gompertz, Richards
Ogunshola et al.	2020	Nigeria	Open Access Journal of Agriculture Research	Fulani	0-182	90	Brody, Gompertz, Logistic, Richards
de Oliveira et al.	2018	Brazil	Semina: ciencias agrarias	Hy-line	0–98	4000	Gompertz, Logistic
Osaiyuwu et al.	2024	Nigeria	Animal Research International	Ross 308	0–40	1286	Gompertz, Logistic, Richards von Bertalanffy
Pinzon et al.	2022	Colombia	Brazilian Journal of Poultry Science	Hy-Line Brown	7-245	15	Gompertz, logistic, Richards von Bertalanffy
Plaengkaew et al.	2021	Thailand	Agriculture and Natural Resources	Thai black bone	0–84	3280	Gompertz, logistic, von Bertalanffy
Rizzi et al.	2013	Italy	Poultry Science	Berlanda B, Padovana, camosciata	1 -180	398	Logistic, Gompertz, Richards
Sakomura et al.	2005	Brazil	Poultry Science	Ross	1 -112	480	Gompertz
Sanusi and Oseni	2020	Nigeria	Genetics and Biodiversity Journal	Fulani	0-140	200	Gompertz, Logistic, Richards, von Bertalanffy
Moroudi et al.	2020	Iran	Journal of agricultural science and Technology	Arian and Urmia crossbreed	0–84	303	Logistic, Richards, von Bertalanffy
Şengül et al.	2024	Turkey	PLOS ONE	RossPM3	0–42		Logistics, Gompertz, Weibull, von Bertalanffy
Setiaji et al.	2023	Indonesia	Journal of the Indonesian Tropical Animal Agriculture	CP 707, Lohman, Cobb, Ross	0–35	1570	Gompertz
Soares et al.	2023	Portugal	Agriculture	Branca	13–195	20	Brody, Gompertz, Logistic
Topal and Bolukbasi	2008	Turkey	Journal of Applied Animal Research	RossPM3	1–42	96	Logistic, Gompertz, von Bertalanffy, Weibull
van der Klein et al.	2020	Canada	Poultry science	Lohmann Brown	0-294	15	Gompertz, Logistic
Xie et al.	2020	China	Animal	Qingyuan Yellow-Feathered	0-119	500	Gompertz, logistic, von Bertalanffy
Zarate-Contreras et al.	2022	Mexico	Poultry Science	Mexican Creole	0-133	286	Logistic, Richards, von Bertalanffy

_ = not stated

### Publication by country

**Fig. 2:**
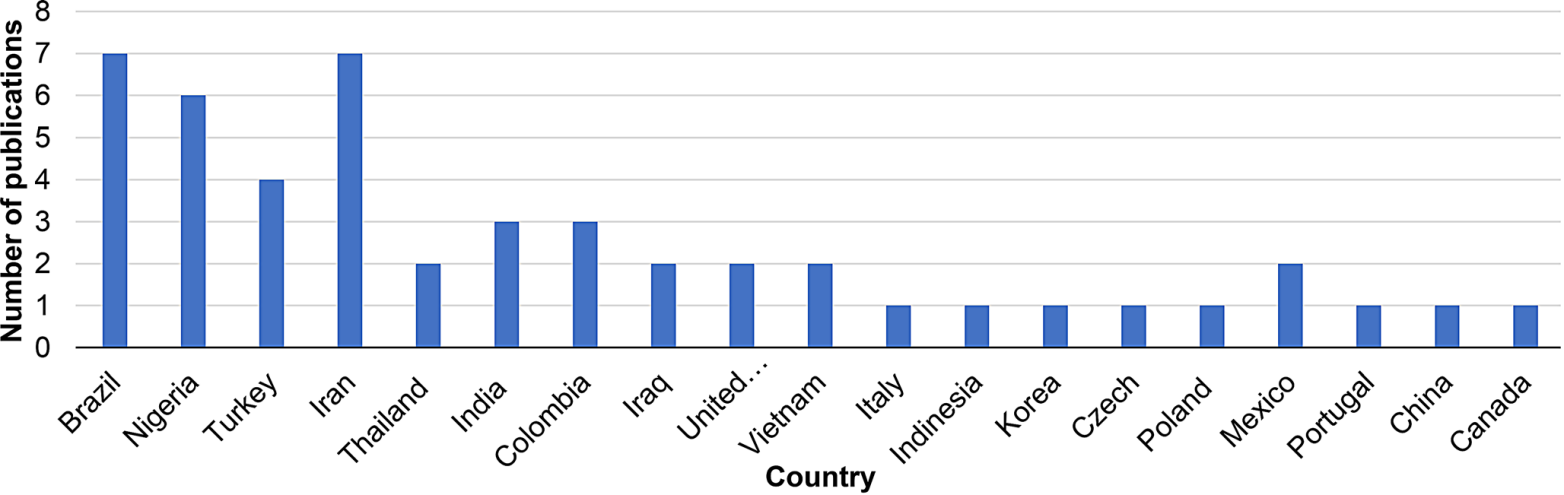
Number of publications by country

Figure 2 shows number of publications by country of 49 included articles. The results revealed that Brazil and Iran had highest number of publications (*n* = 7), followed by Nigeria (*n* = 6). Czech Republic (Knizetova et al. 1995), Italy (Rizzi et al. 2013), Korea (Manjula et al. 2018), Poland (Michalczuk et al. 2016), Portugal (Soares et al. 2023), Indonesia (Setiaji et al. 2023), China (Xie et al. 2020) and Canada (van der Klein et al. 2020) had the least number of publications each (*n* = 1).

### Publication by year

**Fig. 3:**
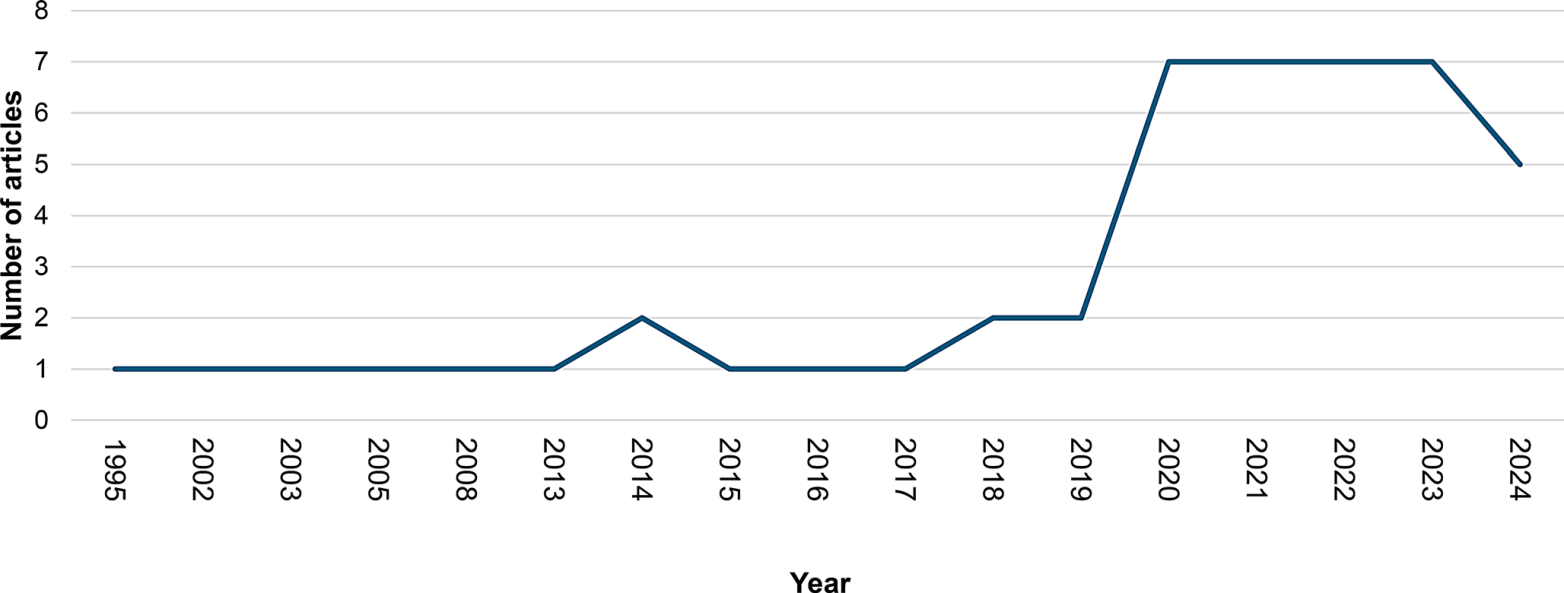
Number of publications by year

Figure 3 shows number of publication by year. The results showed that out of the 49 included articles, the highest number of publications (*n* = 7) occurred on year 2020, 2021, 2022, and 2023. The year, 1995 (Knizetova et al. 1995), 2002 (Aggrey 2002), 2003 (Narushin and Takma 2003), 2005 (Sakomura et al. 2005), 2008 (Topal and Bolukbasi 2008), 2013 (Rizzi et al. 2013), 2015 (Al-Samarai 2015), 2016 (Michalczuk et al. 2016), and 2017 (Masoudi and Azarfar 2017) published least number of articles (*n* = 1).

### Publication by journals

**Fig. 4:**
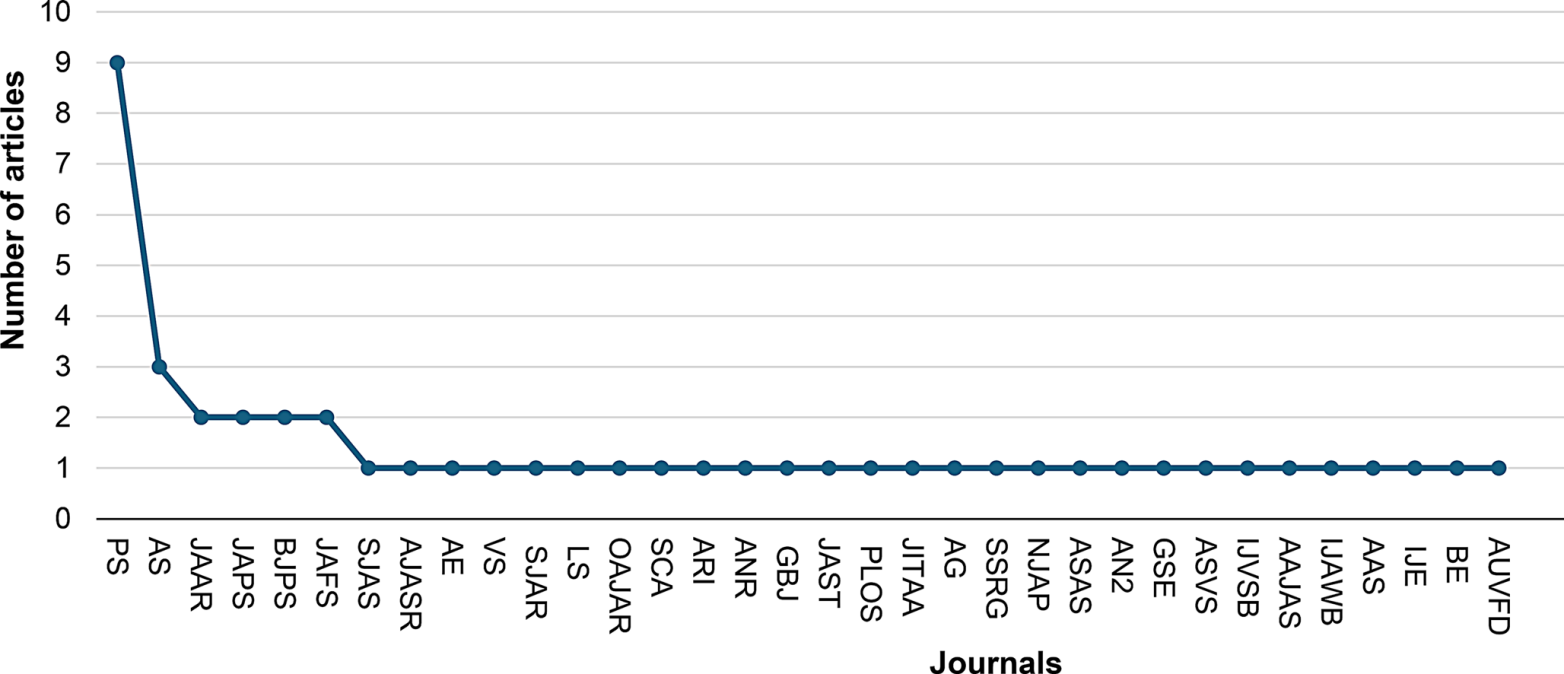
Number of publications by journal. PS = Poultry Science, JAAR = Journal of Applied Animal Research, SJAS = Slovak Journal of Animal Science, AJASR = American Journal of Applied Scientific Research, AE = AgriEngineering, VS = Veterinary Science, SJAR = Spanish Journal of Agricultural Research, BJPS = Brazilian Journal of Poultry Science, LS = Livestock Science, AS = Animal Science, OAJAR = Open Access Journal of Agriculture Research, SCA = Semina: Ciencias Agrarias, ARI = Animal Research International, ANR = Agriculture and Natural Resources, GBJ = Genetics and Biodiversity Journal, JAST = Journal of Agricultural Science and Technology, PLOS = PLOS ONE, JITAA = Journal of the Indonesian Tropical Animal Agriculture, JLST = Journal of Livestock Science and Technology, AG = Agriculture, SSRG = SSRG International Journal of Recent Engineering Science, NJAP = Nigerian Journal of Animal Production, ASAS = Acta Scientiarum Animal Sciences, JAPS = Journal of Animal and Plant Sciences, AN2 = Animals, EPSJ = Egyptian Poultry Science Journal, GSE = Genetics Selection Evolution, ASVS =Acta Scientific Veterinary Sciences, IJVSB = The Indian Journal of Veterinary Sciences and Biotechnology, AAJAS = Asian-Australasian Journal of Animal Sciences, IJAWB = International Journal of Avian and Wildlife Biology, AAS = Annals of Animal Science, IJE = Indian Journal of Ecology, WPS = World’s Poultry Science, BE = Biosystems Engineering, AUVFD = Ankara Universitesi Veteriner Fakultesi Dergisi

Figure 4 shows number of publications by journal. The results indicated that out of 34 published journals, Poultry Science published the highest number of articles (*n* = 9) followed by Animal Science (*n* = 3) and, JAPS, BJPS and JAFS (*n* = 2). The remaining 28 journals had one (1) publication each out of the 49 articles included in this review.

### Publication by chicken breeds

Table 2 shows number of publications by chicken breeds. The results revealed that out of the 48 published chicken breeds, Ross 308 broiler chicken breed was studied by the most articles (*n* = 7). The second most utilized breeds (*n* = 3) were FUNAAB Alpha chicken (Bashiru et al. 2020; Akinsola et al. 2021; Fatai et al. 2023) and Fulani chickens (Ogunshola et al. 2020; Sanusi and Oseni 2020; Akinsola et al. 2021).

**Table 2:** Number of publications by chicken breeds

Chicken breeds	Number of publications	Chicken breeds	Number of publications	Chicken breeds	Number of publications
Lohmann LSL	1	Ross	1	New Francesa	1
Athens Canadian Random Bred	1	Aseel native	2	Hy-line	2
Sasso	1	SaChi	1	Thai black bone	1
Fulani	3	Korean native	1	Berlanda B	1
FUNAAB Alpha	3	Creole	1	Padovana	1
Shika-Brown	1	Medium growing chickens	1	RossPM3	2
Ross 308	7	Iranian native chicken	1	CP 707	1
Broilers	2	Iraqi brown indigenous	1	Lohman	1
Ri Chickens	1	Shaver White	1	Cobb	1
Kai Shee	1	Arian and Urmia crossbreed	2	Ross	2
Kaimook e-san1	1	Fars indigenous	1	Hy Line Brown	1
Color Plume	1	Mexican Creole	1	Lohmann Brown	1
Canela-Preta	1	Kadaknath	1	Isfahan indigenous	1
Cobb500	2	Mia	1	White Leghorn	2
Khazak	1	White Cornish	1	Qingyuan Yellow Feather	1
Yoruba	1	White Plymouth Rock	1	German Langshan bantam	1

### Publication by types of non-linear models

**Fig. 5:**
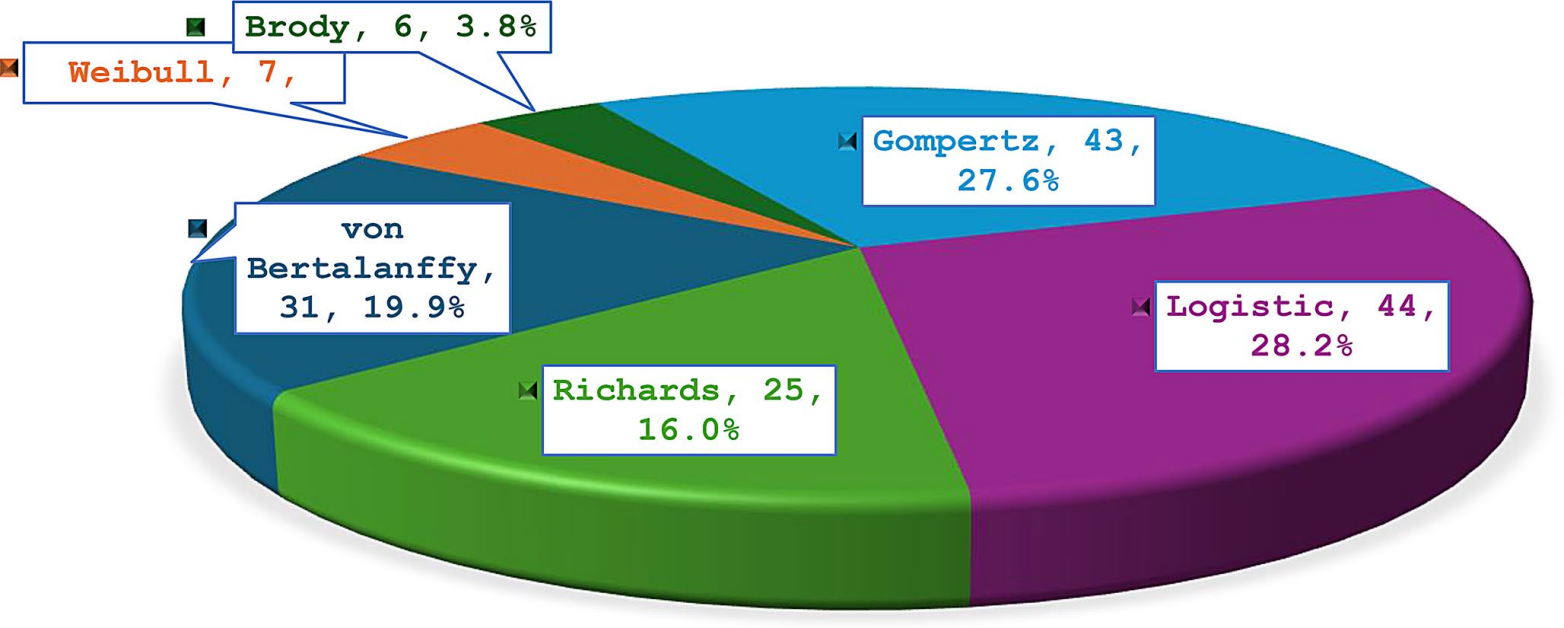
Number of publications by non-linear models

Figure 5 presents number of publications by non-linear models used from the 49 included articles. The results indicated that out of the 6 models published, Logistic model was the most used non-linear model (*n* = 44; 28.2%), followed by Gompertz (*n* = 43; 27.6%), Von Bertalanffy (*n* = 31; 19.9%) and Richards (*n* = 25; 16.0%). Weibull (*n* = 7; 4.5%) and Brody (*n* = 6; 3.8%) were the least studied non-linear models.

### Model fit performance of Gompertz non-linear growth model in chickens

Table 3 shows the fitting performance of estimating growth curve by Gompertz non-linear model applied in 43 articles. The results showed that high accuracies for estimation of growth curves were reported with coefficient of determination ranging from 95% to 100%. The results indicated that Topal and Bolukbasi (2008); Kumar et al. (2022a, b) reported the highest coefficient of determination (R^2^= 1.00). The results indicated that a least coefficient of determination (R^2^ = 0.95) was reported by Mouffok et al. (2019) on Cobb500 chickens and Al-Ali et al. (2022) on Iraqi brown indigenous chickens.

**Table 3:** Model fit performance of Gompertz non-linear growth model in chickens

Author & Year	Breed	Sex	Goodness of fit					
			*R* ^2^	AdjR^2^	MSE	RMSE	AIC	BIC
Aggrey (2002)	Athens Canadian Random Bred	Males	0.9826					
		Females	0.9811					
Akinsola et al. (2021)	Kuroiler	Males		3P-0.996		3P-37.637	122.196	117.121
				4P-0.994		4P-38.624	128.630	118.619
		Females		3P-0.995		3P-33.706	119.769	114.694
				4P-0.992		4P-35.444	126.740	116.729
		Combined		3P-0.998		3P-31.7	118.39	113.32
				4P-0.993		4P-31.84	124.38	114.37
	Sasso	Males		3P-0.996		3P-36.087	121.270	116.195
				4P-0.992		4P-36.146	127.171	117.160
		Females		3P-0.997		3P-48.346	127.704	122.629
				4P-0.992		4P-51.633	135.016	125.006
		Combined		3P-0.995		3P-18.48	106.55	101.47
				4P-0.988		4P-18.94	112.96	102.95
	Fulani	Males		3P-0.996		3P-18.386	101.360	18.386
				4P-0.996		4P-18.912	112.921	102.910
		Females		3P-0.988		3P-13.324	99.351	94.276
				4P-0.992		4P-11.809	102.561	92.550
		Combined		3P-0.996		3P-18.48	106.55	101.47
				4P-0.990		4P-18.94	112.96	102.95
	FUNAAB Alpha	Males		3P-0.994		3P-35.619	120.983	115.908
				4P-0.992		4P-31.100	123.863	113.853
		Females		3P-0.990		3P-43.846	125.555	120.480
				4P-0.989		4P-37.856	128.188	118.177
		Combined		3P-0.987		3P-45.57	126.40	121.33
				4P-0.988		4P-40.66	129.76	119.75
	Shika-Brown	Males		3P-0.996		3P-18.386	107.869	102.794
				4P-0.992		4P-23.033	110.652	100.642
		Females		3P-0.998		3P-28.016	115.702	110.627
				4P-0.995		4P-26.514	120.354	110.343
		Combined		3P-0.998		3P-32.14	103.44	91.65
				4P-0.978		4P-29.24	112.96	102.95
	Noiler	Males		3P-0.997		3P-22.119	89.819	76.804
				4P-0.994		4P-16.697	102.202	72.599
		Females		3P-0.992		3P-58.794	105.461	92.445
				4P-0.989		4P-59.162	1222.442	92.839
		Combined		3P-0.996		3P-45.17	101.24	88.22
				4P-0.996		4P-50.30	119.84	90.24
Al-Samarai (2015)	Ross 308	Males	0.997	0.989	497.6			
Bashiru et al. (2020)	FUNAAB Alpha	Males					50.42	61.528
		Females					44.460	55.102
Benvenga et al. (2022)	Broilers		0.999998					
Bo et al. (2022)	Ri chickens	Males	0.9725				40,328	40352.3
		Females	0.9724				37948.1	37972.5
Boonkum et al. (2021)	Kai Shee	Males			54,722			
		Females			17,519			
		Combined			37,948			
	Kaimook e-san1	Males			46,195			
		Females			33,243			
		Combined			47,047			
Brito et al. (2021)	Color Plume			0.999				
de Figueiredo et al. 2023	Athens -Canadian Random Bred	Males		0.9993		430.83	254.13	259.46
Falana et al. (2024)	Cobb500	Males	0.99			273.00		
		Females	0.99			203.25		
Faraji-Arough et al. (2019)	Khazak	Males		0.9030		125.01	16622.89	16643.66
		Females		0.9009		98.56	21170.93	21192.83
Fatai et al. (2023)	Yoruba	–	0.998				60.480	58.670
	FUNAAB Alpha		0.996				88.150	86.350
de Freitas et al. (2023)	Cobb500	Males	0.99					
Galeano-Vasco et al. (2014)	Lohmann LSL layers	Females					8419.4	8428.2
Gautam (2024)	Kadaknath	Males	0.9955	0.9946	571.30	23.90	91.49	93.41
		Females	0.9962	0.9955	411.88	20.29	86.91	88.83
Ghaderi-Zefrehei et al. (2024)	Isfahan indigenous chicken		0.9901		20784.9		209922.1	
Kumar et al. (2022a)	Aseel native chicken	Males	1.000	1.000	146.945		19.36	
		Females	0.999	0.999	163.725		17.875	
		Combined	0.999	0.999	152.118		18.603	
Kumar et al. (2022a, b)	Aseel native chicken	Males	0.997	0.998	469.92		29.839	
		Females	0.996	0.984	237.19		30.555	
		Combined	0.996	0.984	331.565		30.004	
Machado et al. (2023)	Canela-Preta		0.997		1697.5333			
Manjula et al. (2018)	Korean native chicken	Combined	0.9955					
Masoudi and Azarfar (2017)	Ross 308	Combined	0.9970				648	
Michalczuk et al. (2016)	Medium growing chickens	Males	0.9963	0.9944				
		Females	0.9982	0.9973				
Moharrery and Mirzaei (2014)	Ross 308	–	0.9914					
	Iranian native chickens		0.9879					
Mouffok et al. (2019)	Cobb500		0.954		51645.2	227.3	81.96	
Al-Ali et al. (2022)	Iraqi brown indigenous chickens	Males	0.95		15894.4		-704.8	266.7
		Females	0.95		65013.9		730.8	2774.9
Narushin and Takma (2003)	Shaver White laying hens	Females	0.999					
Nematzadeh et al. (2022)	Arian	Males		0.998			78.2	
		Females		0.997			65.5	
Neysi et al. (2023)	Fars indigenous chicken		0.977484		22356.8	149.5219	143997.54	201786.49
Hoang et al. (2021)	Mia chickens	Males					34,706	34,729
		Females					24,667	24,690
Ogunshola et al. (2020)	Fulani	Males	0.998		2218.9			
		Females	0.999		1138.5			
de Oliveira et al. (2018)	Hy-line	–	0.999		50,806		9.52	
Osaiyuwu et al. (2024)	Ross 308			0.995587		62.88		
Pinzon et al. (2022)	Hy Line Brown	Females		0.999	-	-	306.058	312.393
Plaengkaew et al. (2021)	Thai black bone	Males	0.93 ± 0.01			79.8 ± 2.7		
		Females	0.94 ± 0.01			69.3 ± 3.1		
		Combined	0.93 ± 0.01			110.1 ± 4.7		
Rizzi et al. (2013)	Berlanda B,	–	0.9987			41		
	Padovana,		0.9979			36		
	Camosciata		0.9937			38		
Sakomura et al. (2005)	Ross	Males	0.99					
		Females	0.99					
Sanusi and Oseni (2020)	Fulani	Males	0.9957	0.9831	3916.20		173.47	190.35
		Females	0.9957	0.9831	3916.20		173.46	190.35
Şengül et al. (2024)	RossPM3	Females	0.9998	0.9997	470.570	21.681	68.750	68.934
Setiaji et al. (2023)	CP 707	–	0.999				-224.96	
	Lohman		0.999				-247.935	
	Cobb		0.999				-193.167	
	Ross		0.999				-199.838	
Soares et al. (2023)	Branca	Males	0.99			3162		
		Females	0.99			13.384		
Topal and Bolukbasi (2008)	RossPM3	Males	0.996		1827.27			
		Females	1.000		263.766			
van der Klein et al. (2020)	Lohmann Brown	Females	0.986			0.103		
Xie et al. (2020)	QingyuanYellow-Feathered				13.611		108.801	109.994

R^2^ = coefficient of determination, AdjR^2^ = adjusted coefficient of determination, MSE = mean square error, _ = not stated, RMSE = residual mean square error, AIC = Akaike information criterion, BIC = Bayesian information criterion, 3P = 3-parameter model, 4P = 4-parameter model

### Model fit performance of Logistic non-linear growth model in chickens

Table 4 shows fitting performance of estimating growth curve of chickens using Logistic non-linear model. The results indicated that the Logistic model was applied in 44 out of 49 articles included, and that the coefficient of determination ranged from 16.3% to 99.9%. The results indicated that all the articles reported a strong model fit with a coefficient of determination of R^2^ ≥ 0.95, however, Ogunshola et al. (2020) reported the least coefficient of determination (R^2^ = 0.163) on Fulani chickens.

**Table 4:** Model fit performance of Logistic non-linear growth model in chickens

Author & Year	Breed	Sex	Goodness of fit					
			*R* ^2^	AdjR^2^	MSE	RMSE	AIC	BIC
Aggrey (2002)	Athens Canadian Random Bred	Males	0.9800					
		Females	0.9780					
Akinsola et al. (2021)	Kuroiler	Males		3P-0.990		3P-54.721	130.429	125.354
				4P-0.989		4P-44.523	131.732	121.721
		Females		3P-0.990		3P-47.074	132.982	122.972
				4P-0.985		4P-74.792	137.304	132.228
		Combined		3P-0.992		3P-73.29	129.70	119.69
				4P-0.993		4P-40.56	136.85	131.78
	Fulani	Males		3P-0.992		3P-23.033	111.393	106.318
				4P-0.992		4P-19.302	113.370	103.359
		Females		3P-0.992		3P-11.183	90.350	80.340
				4P-0.995		4P-6.779	95.497	90.422
		Combined		3P-0.996		3P-20.61	106.55	101.47
				4P-0.993		4P-15.96	109.19	99.18
	FUNAAB Alpha	Males		3P-0.994		3P-31.158	118.040	112.964
				4P-0.994		4P-27.272	120.974	110.963
		Females		3P-0.994		3P-37.019	121.831	116.756
				4P-0.990		4P-35.482	126.763	118.177
		Combined		3P-0.994		3P-33.26	119.47	114.40
				4P-0.989		4P-31.34	124.03	114.02
	Sasso	Males		3P-0.992		3P-53.756	130.038	124.963
				4P-0.990		4P-41.523	130.222	120.211
		Females		3P-0.988		3P-80.110	138.815	133.740
				4P-0.987		4P-63.726	139.646	129.635
		Combined		3P-0.992		3P-20.61	108.94	103.87
				4P-0.989		4P-15.96	109.19	99.18
	Noiler	Males		3P-0.995		3P-34.954	97.141	84.125
				4P-0.994		4P-16.666	102.172	72.569
		Females		3P-0.990		3P-69.867	108.221	95.206
				4P-0.989		4P-58.948	122.384	92.781
		Combined		3P-0.993		3P-48.23	102.29	89.27
				4P-0.996		4P-48.63	119.30	89.70
	Shika- Brown	Males		3P-0.998		3P-19.624	107.869	102.794
				4P-0.995		4P-17.059	110.652	100.642
		Females		3P-0.998		3P-29.213	116.621	111.546
				4P-0.998		4P-24.756	118.844	108.833
		Combined		3P-0.993		3P-48.23	104.67	93.89
				4P-0.996		4P-48.63	122.30	94.32
Al-Samarai (2015)	Ross 308	Males	0.981	0.994	497.6			
Bashiru et al. (2020)	FUNAAB Alpha	Males					53.23	64.488
		Females					47.10	58.342
Benvenga et al. (2022)	Broilers		0.999					
Bo et al. (2022)	Ri chickens	Males	0.9689				40759.5	40735.2
		Females	0.967				38464.7	38489.1
Boonkum et al. (2021)	Kai Shee	Males			55,044			
		Females			17,703			
		Combined			32,206			
	Kaimook e-san1	Males			46,256			
		Females			33,412			
		Combined			47,311			
de Figueiredo et al. (2023)	Athens Canadian Random Bred	Males		0.9966		2113.96	298.66	303.99
Falana et al. (2024)	Cobb500	Males	0.99			244		
		Females	0.99			230.28		
Faraji-Arough et al. (2019)	Khazak	Males		0.8981		128.14	16688.70	16709.47
		Females		0.8935		102.17	21297.53	21319.43
Fatai et al. (2023)	Yoruba FUNAAB Alpha		0.998				62.71	60.890
			0.992				97.470	95.66
Gautam (2024)	Kadaknath	Males	0.9933	0.9921	836.09	28.92	96.83	98.74
		Females	0.9949	0.9940	546.88	23.39	90.88	88.83
Ghaderi-Zefrehei et al. (2024)	Isfahan indigenous chicken		0.9855		23165.9		211214.3	
Quintana-Ospina et al. (2023)	Ross 308	Males	0.995			44.7	6834.7	6852.6
		Females	0.994			44.9	7968.6	7987.2
Kucuktopcu and Cemek (2021)	Ross 308					36.28		
Kumar et al. (2022a)	Aseel native chicken	Males	0.999	0.999	276.672		19.992	
		Females	0.999	0.999	143.470		18.495	
		Combined	0.999	0.999	108.820		19.236	
Kumar et al. (2022a, b)	Aseel native chicken	Males	0.999	0.999	143.470		32.862	
		Females	0.999	0.999	276.672		29.734	
		Combined	0.999	0.999	200.481		31.323	
Machado et al. (2023)	Canela-Preta	–	0.993		5554.933			
Manjula et al. (2018)	Korean native chicken	Combined	0.9957					
Masoudi and Azarfar (2017)	Ross 308	Combined	0.9960				672	
Mata-Estrada et al. (2020)	Creole	Males	0.9360				2440.9	2450.4
		Females	0.9305				2243.9	2253.3
Michalczuk et al. (2016)	Medium growing chickens	Males	0.9928	0.9892				
		Females	0.9954	0.9930				
Moharrery and Mirzaei (2014)	Ross 308			0.9945				
	Iranian native chickens			0.9900				
Mouffok et al. (2019)	Cobb500	–	0.954		52181.2	227.3	82.04	
Al-Ali et al. (2022)	Iraqi brown indigenous chickens	Males	0.95		155355.7		-701.1	2870.3
		Females	0.95		63716.6		-722.7	2783.0
Narushin and Takma 2003	Shaver White laying Hens	Females	0.995					
Nematzadeh et al. (2022)	Arian	Males		0.975			68.62	
		Females		0.995			70.42	
Neysi et al. (2023)	Fars indigenous chicken		0.977314		22524.8	150.0826	144063.16	201937.6
Hoang et al. (2021)	Mia chickens	Males					34,844	34,867
		Females					24,921	24,943
Ogunshola et al. (2020)	Fulani	Males	0.998		2218.9			
		Females	0.163		1169.8			
de Oliveira et al. (2018)	Hy-line		0.95		102,468		10.22	
Osaiyuwu et al. (2024)	Ross 308			0.995151		65.92		
Pinzon et al. (2022)	Hy Line Brown	Females		0.999			383.566	389.899
Plaengkaew et al. (2021)	Thai black bone	Males	0.93 ± 0.01			111.8 ± 5.1		
		Females	0.93 ± 0.01			114.9 ± 4.5		
		Combined	0.93 ± 0.01			113.1 ± 3.9		
Rizzi et al. (2013)	Berlanda B		0.9971			64		
	Padovana		0.9967			46		
	Camosciata		0.9918			57		
Sanusi and Oseni (2020)	Fulani	Males	0.9921	0.9689	1567.06		195.38	204.88
		Females	0.9922	0.9689	1567.06		195.38	204.88
Moroudi et al. (2020)	Arian and Urmia crossbreed	Males	0.977875	0.977833		0.269873	344.847	366.249
		Females	0.980467	0.980439		0.201972	-753.163	-730.568
Şengül et al. (2024)	RossPM3	Males	0.9997	0.9995	629.327	25.086	70.357	70.802
Soares et al. (2023)	Branca	Males	0.95			3862		
		Females	0.97			21,366		
Topal and Bolukbasi (2008)	RossPM3	Males	0.996		1827.27			
		Females	0.998		995.740			
van der Klein et al. (2020)	Lohmann Brown	Females	0.986			0.105		
Xie et al. (2020)	QingyuanYellow-Feathered					21.992	113.783	114.977
Zarate-Contreras et al. (2022)	Mexican Creole			0.94484			50843.27	50868.39

R^2^ = coefficient of determination, AdjR^2^ = adjusted coefficient of determination, MSE = mean square error, _ = not stated, RMSE = residual mean square error, AIC = Akaike information criterion, BIC = Bayesian information criterion, 3P = 3-parameter model, 4P = 4-parameter model

### Model fit performance of von Bertalanffy non-linear growth model in chickens

Table 5 shows fitting performance of estimating growth curve of chickens using von Bertalanffy non-linear model in 31 out of 49 included articles. The results indicated that the coefficient of determination ranged from 93.8% to 99.9%.

**Table 5:** Model fit performance of von Bertalanffy non-linear growth model in chickens

Author & Year	Breed	Sex	Goodness of fit					
			*R* ^2^	Adj^2^	MSE	RMSE	AIC	BIC
Bashiru et al. (2020)	FUNAAB Alpha	Males					49.42	60.122
		Females					44.21	54.154
Benvenga et al. (2022)	Broilers		0.999739					
Bo et al. (2022)	Ri chickens	Males	0.9722				40364.1	40388.4
		Females	0.9728				37907.08	37931.43
Boonkum et al. (2021)	Kai Shee	Males			54,871			
		Females			17,621			
		Combined			38,083			
	Kaimook e-san1	Males			47,033			
		Females			33,891			
		Combined			47,851			
de Figueiredo et al. (2023)	Athens -Canadian Random Bred	Males		0.9984		999.76	277.70	283.03
Falana et al. (2024)	Cobb500	Males	0.97			273.00		
		Females	0.98			203.25		
Faraji-Arough et al. (2019)	Khazak	Males		0.9031		124.91	16621.81	16647.77
		Females		0.9025		97.74	21142.48	21169.85
Fatai et al. (2023)	Yoruba		0.999				85.360	83.550
	FUNAAB Alpha		0.995				83.720	81.910
Quintana-Ospina et al. (2023)	Ross 308	Males	0.996			41.5	6737.4	6755.4
		Females	0.996			41.7	7854.2	7872.7
Gautam (2024)	Kadaknath	Males	0.9952	0.9943	598.46	24.40	92.14	94.06
		Females	0.9953	0.9944	506.25	22,50	89.80	91.72
Ghaderi-Zefrehei et al. (2024)	Isfahan indigenous chicken		0.9868		21004.2		210130.5	
Galeano-Vasco et al. (2014)	Lohmann LSL layers	Females					8478.3	8487.1
Kucuktopcu and Cemek (2021)	Ross 308					27.71		
Kumar et al. (2022a)	Aseel native chicken	Males	0.999	0.999	276.672		19.129	
		Females	0.999	0.999	143.470		17.645	
		Combined	0.999	0.999	108.820		18.368	
Kumar et al. (2022a, b)	Aseel native chicken	Males	0.999	0.999	398.7150		34.756	
		Females	0.998	0.997	4111.360		34.607	
		Combined	0.999	0.999	401.072		34.635	
Machado et al. (2023)	Canela-Preta		0.998		1312.02			
Manjula et al. (2018)	Korean native chicken	Combined	0.9945					
Mata-Estrada et al. (2020)	Creole	Males	0.9415				2424.8	2434.3
		Females	0.9380	-	-	-	2224.1	2233.5
Mouffok et al. (2019)	Cobb500		0.954		52420.8	228.9	82.07	
Al-Ali et al. (2022)	Iraqi brown indigenous chickens	Males	0.95		154266.6		-702.2	2868.6
		Females	0.95		64543.7		-727.7	2778.0
Narushin and Takma (2003)	Shaver White laying Hens	Females	0.999					
Nematzadeh et al. (2022)	Arian	Males		0.998			83.94	
		Females		0.999			76.14	
Osaiyuwu et al. (2024)	Ross 308			0.995573		62.98		
Pinzon et al. (2022)	Hy Line Brown	Females		0.999			327.388	333.722
Plaengkaew et al. (2021)	Thai black bone	Males	0.94 ± 0.01			1109 ± 4.1		
		Females	0.94 ± 0.01			94.0 ± 4,6		
		Combined	0.93 ± 0.00			109.2 ± 4.1		
Sanusi and Oseni (2020)	Fulani	Males	0.9965	0.9862	4111.99		176.04	185.47
		Females	0.9965	0.9862	4111.99		176.04	185.47
Moroudi et al. (2020)	Arian and Urmia crossbreed	Males	0.978607	0.978552		0.265457	294.463	321.216
		Females	0.981381	0.981345		0.197236	-851.719	-823.475
Şengül et al. (2024)	RossPM3	Females	0.9996	0.9994	821.485	28.662	72.317	72.829
Topal and Bolukbasi (2008)	RossPM3	Males	0.998		731.821			
		Females	0.999		318.282			
Xie et al. (2020)	QingyuanYellow-Feathered					11.308	106.457	107.651
Zarate-Contreras et al. (2022)	Mexican Creole			0.94309			51039.07	51064.19

R^2^ = coefficient of determination, AdjR^2^ = adjusted coefficient of determination, MSE = mean square error, _ = not stated, RMSE = residual mean square error, AIC = Akaike information criterion, BIC = Bayesian information criterion

### Model fit performance of Richards non-linear growth model in chickens

Table 6 shows fitting performance of estimating growth curve of chickens using Richards non-linear model. The results showed that Richards model was applied in 25 out of 49 articles included, and that coefficient of determination ranged from 16.3% to 99.8%. Most articles reported a strong model fit with a coefficient of determination (R^2^ ≥ 0.938). However, Ogunshola et al. (2020) reported a low coefficient of determination (R^2^ = 0.163) on female Fulani chickens.

**Table 6:** Model fit performance of Richards non-linear growth model in chickens

Author & Year	Breed	Sex	Goodness of fit					
			*R* ^2^	AdjR^2^	MSE	RMSE	AIC	BIC
Aggrey (2002)	Athens Canadian Random Bred	Males	0.9827					
		Females	0.9812					
Bashiru et al. (2020)	FUNAAB Alpha	Males					50.52	61.778
		Females					46.76	57.813
Bo et al. (2022)	Ri chickens	Males	0.9725				40,330	40360.5
		Females	0.9728				37906.1	37936.5
de Figueiredo et al. (2023)	Athens -Canadian Random Bred	Males		0.9993		456.78	256.62	263.28
Faraji-Arough et al. (2019)	Khazak	Males		0.9031		124.94	16622.39	16648.35
		Females		0.9024		97.82	21145.20	21172.56
Galeano-Vasco et al. (2014)	Lohmann LSL layers	Females					8424.0	8434.1
Gautam (2024)	Kadaknath	Males	0.9954	0.9940	628.94	25.08	93.51	96.06
		Females	0.9963	0.9952	439.02	20.95	88.47	91.03
Knizetova et al. (1995)	White Cornish, White Plymouth Rock		0.9976					
Kucuktopcu and Cemek (2021)	Ross 308					27.62		
Machado et al. (2023)	Canela-Preta		0.998		944.398			
Masoudi and Azarfar (2017)	Ross 308	Combined	0.9970				655	
Mata-Estrada et al. (2020)	Creole	Males	0.9414				2426.5	2439.2
		Females	0.9382				2226.1	2238.6
Michalczuk et al. (2016)	Medium growing chickens	Males	0.9962	0.9931				
		Females	0.9981	0.9966				
Moharrery and Mirzaei (2014)	Ross 308			0.9951				
	Iranian native chickens			0.9912				
Mouffok et al. (2019)	Cobb500		0.954		51658.0	227.3	83.97	
Narushin and Takma (2003)	Shaver White laying Hens		0.982					
Nematzadeh et al. (2022)	Arian	Males		0.999			67.8	
		Females		1.00			54.2	
Hoang et al. (2021)	Mia chickens	Males					34,708	34,737
		Females					24,669	24,697
Ogunshola et al. (2020)	Fulani	Males	0.984		4140			
		Females	0.163		1107.2			
Osaiyuwu et al. (2024)	Ross 308	–		0.994181		0.7221		
Pinzon et al. (2022)	Hy Line Brown	Females		0.999			305.838	313.756
Rizzi et al. (2013)	Berlanda B		0.9991			36		
	Padovana		0.9987			28		
	Camosciata		0.9927			33		
Sanusi and Oseni (2020)	Fulani	Males	0.9922	0.9690	1744.80		195.40	208.06
		Females	0.9923	0.9690	1744.80		195.40	208.06
Moroudi et al. (2020)	Arian and Urmia crossbreed	Males	0.978607	0.978552		0.265457	294.463	321.216
		Females	0.981381	0.981345		0.197236	-851.719	-823.475
Zarate-Contreras et al. (2022)	Mexican Creole		0.94510				50897.21	50922.33

R^2^ = coefficient of determination, AdjR^2^ = adjusted coefficient of determination, MSE = mean square error, _ = not stated, RMSE = residual mean square error, AIC = Akaike information criterion, BIC = Bayesian information criterion

### Model fit performance of Weibull non-linear growth model in chickens

Table 7 shows fitting performance of estimating growth curve of chickens by Weibull model. The results indicated that the Weibull model was used in 7 out of 49 articles included, and that accuracy of predicting growth curves of chickens ranged from 95% to 100%. Topal and Bolukbasi (2008) reported the highest accuracy of prediction (R² = 1.000) on male RossPM3 chickens. Mouffok et al. (2019) reported the least coefficient of determination (R² = 0.999) on Cobb500 chickens.

**Table 7:** Model fit performance of Weibull non-linear growth model in chickens

Author & Year	Breed	Sex	Goodness of fit					
			*R* ^2^	Adj *R*^2^	MSE	RMSE	AIC	BIC
Ghaderi-Zefrehei et al. (2024)	Isfahan indigenous chicken	–	0.9732			21850.2	215210.4	
Moharrery and Mirzaei (2014)	Ross 308			0.994				
	Iranian native chickens			0.9879				
Mouffok et al. (2019)	Cobb500		0.953		53076.6	230.4	84.15	
Narushin and Takma (2003)	Shaver White laying Hens	–	0.999					
Neysi et al. (2023)	Fars indigenous chicken		0970659		23491.8		1444893.93	203855.8
Şengül et al. (2024)	RossPM3	Males	0.9996	0.9993	1085.258	32.943	74.723	75.117
Topal and Bolukbasi (2008)	RossPM3	Males	0.999		646.394			
		Females	1.000		198.515			

R^2^ = coefficient of determination, AdjR^2^ = adjusted coefficient of determination, MSE = mean square error, _ = not stated, RMSE = residual mean square error, AIC = Akaike information criterion, BIC = Bayesian information criterion

### Model fit performance of Brody non-linear growth model in chickens

Table 8 shows fitting performance of estimating growth curve of chickens using Brody model non-linear model used in 6 out of 49 articles included. The results indicated that coefficient of determination ranged from 95.0% to 99.0%. A high accuracy of prediction (R^2^ = 0.99) was reported by Ghaderi-Zefrehei et al. (2024) on Isfahan indigenous chicken, Ogunshola et al. (2020) on female Fulani chickens, and Soares et al. (2023) on female Branca chickens. Al-Ali et al. (2022) reported a least coefficient of determination (R^2^ = 0.95) on Iraqi brown indigenous chickens.

**Table 8:** Model fit performance of Brody non-linear growth model in chickens

Author & Year	Breed	Sex		Goodness of fit				
			*R* ^2^	AdjR^2^	MSE	RMSE	AIC	BIC
de Figueiredo et al. (2023)	Athens -Canadian Random Bred	Males		0.9865		8301.91	256.62	263.28
Ghaderi-Zefrehei et al. (2024)	Isfahan indigenous chicken		0.9844	-	24690.8		212223.7	
Al-Ali et al. (2022)	Iraqi brown indigenous chickens	Males	0.95		221409.5		-616.2	2955.0
		Females	0.95		75266.1		-691.0	75266.1
Neysi et al. (2023)	Fars indigenous chicken		0.972454		27351.1	165.3817	145761.64	205848.47
Ogunshola et al. (2020)	Fulani	Males	0.984		4140			
		Females	0.99		1138.5			
Soares et al. (2023)	Branca	Males	0.98			275		
		Females	0.99			6265		

R^2^ = coefficient of determination, AdjR^2^ = adjusted coefficient of determination, MSE = mean square error, _ = not stated, RMSE = residual mean square error, AIC = Akaike information criterion, BIC = Bayesian information criterion

## Discussion

Growth curves in chickens are one of the most important tools in poultry production to optimize the slaughter age and minimize feed supply cost (Hoang et al. 2021). Accurate model estimation assists in determining optimal growth rates and maturity ages, which are essential for efficient feed utilization by reduced production costs (de Oliveira et al. 2018). This systematic review was conducted to document the performance of various non-linear models in describing the growth curves of chickens and to identify the models that provide the best fit for predicting growth traits. The findings of this systematic review showed that forty-nine articles were included after the screening for eligibility. The results showed that highest number of publications were recorded each year from 2020 to 2023. Brazil and Iran were countries with most articles published and the most studied breed was Ross 308 chicken. Logistic model was the most utilized non-linear model, followed by Gompertz non-linear model. Akinsola et al. (2021) reported that different breeds of chickens grow at different rates or patterns. The results indicated that all reported non-linear models (Gompertz, Logistic, Brody, von Bertalanffy, Richards, Weibull) in articles included explained at least 70% variability in body weight of the chickens (Ogunshola et al. 2020; Neysi et al. 2023; Ghaderi-Zefrehei et al. 2024), thus they are good at estimating growth curves of chickens. Topal and Bolukbasi (2008) on RossPM3 females and Kumar et al. (2022) on male Assel native reported that Gompertz model explained 100% of variation in body weight of chickens when estimating growth curves. Topal and Bolukbasi (2008) reported the highest variability in body weight of 100% on RossPM3 chickens by Weibull model models. Perfect fitting by Gompertz model could have resulted from modeling initial scaling weight data at 50% of the asymptotic weight, while Weibull model is very flexible to explain variation in initial scaling weight at 30% of the asymptotic weight. The reason for having a coefficient of determination of 100% (perfect fit) may be due to the models representing a true relationship of the change in body weight of chickens over certain points in time (growth). However, overfitting could occur when a model does not explain well from testing (observed) data to the training (unseen) data. This could causes false accuracy of estimating growth curves in chickens using testing data. The results imply that Gompertz, and Weibull non-linear models may be best suitable models for estimating the change in chickens’ body weight over time (growth curves). The strength of this systematic review is that no similar study was conducted on the estimation of growth curves on chickens using non-linear models. Thus, there is no comparison of results on the current study with other studies. The major contribution of this systematic review to the body of knowledge is that it evaluates available evidence on the performance of different non-linear models and provides a scientific basis for model selection in chicken production. The limitations are that some articles failed to report key goodness-of-fit statistics, such as root mean square error, Akaike information criterion, the model parameter used, and Bayesin information criterion while others combined male and female results which reduces accuracy of prediction for growth curves of each sex.

## Conclusion

Non-linear models in the current study can be employed in estimation of growth curves to understand the growth patterns of chickens. Moreover, Gompertz and Weibull non-linear models outperformed others included non-linear models and this makes them more reliable in estimating growth curves of chickens. The results can assist future researchers in selecting best models for estimating the growth curves parameters of chickens. It may assist breeders in selection of best breed or lines of chickens that exhibit superior growth performance at early stages for breeding purposes. This can promote efficient feed utilization and faster growth rate to meet the market weights. Researchers should include root mean square error, Akaike information criterion, Bayesin information criterion, and employ a larger sample size to increase the accuracy of predicting growth curves of chickens. There is a need to ensure that larger data with less outliers, and the use of suitable parameter model on a compatible data set is used to avoid overfitting and high false accuracy.

## Data Availability

The articles that were painstakingly searched are listed and tabulated in this study and the corresponding author (Thobela Louis Tyasi) has copies of the articles.
